# Cerebral Veins and Dural Sinuses Thrombosis: Data from a Monocentric Cohort Study of 55 Romanian Patients

**DOI:** 10.3390/life16060875

**Published:** 2026-05-23

**Authors:** Dragos Catalin Jianu, Silviana Nina Jianu, Razvan Bertici, Nicoleta Iacob, Sergiu Florin Arnautu, Georgiana Munteanu, Traian Flavius Dan, Raphael Sadik, Tudor Nicolae Jianu, Any Axelerad, Cristina Tudoran, Andrei Gheorghe Marius Motoc, Stefan Strilciuc, Dafin Fior Muresanu, Ligia Petrica

**Affiliations:** 1 First Division of Neurology, Department of Neurosciences, Victor Babes University of Medicine and Pharmacy, E. Murgu Sq., No. 2, 300041 Timisoara, Romania; jianu.dragos@umft.ro (D.C.J.); munteanu.georgiana@umft.ro (G.M.); traian.dan@umft.ro (T.F.D.); tudor.jianu@student.umft.ro (T.N.J.); 2 Centre for Cognitive Research in Neuropsychiatric Pathology (NeuroPsy-Cog), Department of Neurosciences, Victor Babes University of Medicine and Pharmacy, 156 L. Rebreanu Ave., 300736 Timisoara, Romania; arnautu.sergiu@umft.ro (S.F.A.); docuaxi@yahoo.com (A.A.); tudoran.cristina@umft.ro (C.T.); amotoc@umft.ro (A.G.M.M.); petrica.ligia@umft.ro (L.P.); 3 First Department of Neurology, Pius Brînzeu Clinical Emergency County Hospital, 156 L. Rebreanu Ave., 300736 Timisoara, Romania; 4 Centre for Molecular Research in Nephrology and Vascular Pathology, Department of Internal Medicine II, Victor Babes University of Medicine and Pharmacy, 156 L. Rebreanu Ave., 300736 Timisoara, Romania; 5 Department of Ophthalmology, Dr. Victor Popescu Military Emergency Hospital, 7 G. Lazar Ave., 300080 Timisoara, Romania; silvianajianu@yahoo.com; 6 Department of Multidetector Computed Tomography and Magnetic Resonance Imaging, Neuromed Diagnostic Imaging Centre, 300218 Timisoara, Romania; nicoiacob@yahoo.co.uk; 7 Department of Internal Medicine I, Victor Babes University of Medicine and Pharmacy, E. Murgu Sq., No. 2, 300041 Timisoara, Romania; 8 Department of Geriatrics-Rehabilitation, Riviera-Chablais Hospital, 3 Prairie Ave., 1800 Vevey, Switzerland; raphael.sadik@hopitalrivierachablais.ch; 9 Department of Neurology, General Medicine Faculty, Ovidius University, 900527 Constanta, Romania; 10 Discipline of Cardiology, Department VII-Internal Medicine II, Victor Babes University of Medicine and Pharmacy, E. Murgu Sq., No. 2, 300041 Timisoara, Romania; 11 Department of Anatomy and Embryology, Victor Babes University of Medicine and Pharmacy, E. Murgu Sq., No. 2, 300041 Timisoara, Romania; 12 Research Center for Functional Genomics, Biomedicine and Translational Medicine, Iuliu Haţieganu University of Medicine and Pharmacy, 32-38 Gheorghe Marinescu St., 400347 Cluj-Napoca, Romania; 13 Department of Neurosciences, Iuliu Hațieganu University of Medicine and Pharmacy, No. 8 Victor Babeș Street, 400012 Cluj-Napoca, Romania; dafinm@ssnn.ro; 14 Division of Nephrology, Department of Internal Medicine II, Victor Babes University of Medicine and Pharmacy, E. Murgu Sq., No. 2, 300041 Timisoara, Romania

**Keywords:** cerebral venous sinus thrombosis, stroke, thrombophilia, neuroimaging, anticoagulation, clinical outcomes

## Abstract

Cerebral venous sinus thrombosis (CVST) is a rare form of stroke with heterogeneous clinical presentation and etiological profile. This study aimed to evaluate the demographic, clinical, imaging, etiological, treatment, and outcome characteristics of CVST in a Romanian population. We conducted a retrospective monocentric cohort study including 55 patients diagnosed over a six-year period in a tertiary neurological center. CVST accounted for 1.2% of all stroke cases, with an incidence of 13.75 per million per year. Gender distribution was relatively balanced, differing from international cohorts, and traditional female-specific risk factors were less prominent. Thrombophilia (68.6%) and infections (38.2%) were the most frequent etiologies. Younger patients were more frequently associated with thrombophilia, while a higher inflammatory response was observed in older individuals; additionally, female patients showed a higher prevalence of the MTHFR C677T mutation. Transverse and sigmoid sinuses were the most affected, while cavernous sinus involvement was more frequent than typically reported and associated with infections. At discharge, long-term anticoagulation was recommended in 67.3% of patients, tailored to underlying etiology and risk profile. Outcomes were favorable, with 70–80% achieving a modified Rankin Scale score ≤2 at discharge and over 90% at 90-day follow-up, despite inconsistent correlation with imaging findings. These results highlight the heterogeneous nature of CVST and the need for comprehensive, individualized evaluation and management.

## 1. Introduction

Cerebral venous sinus thrombosis (CVST) is a relatively rare form of stroke, accounting for approximately 0.5–3% of all stroke cases, with an estimated incidence of 1–12 per million individuals per year. However, recent data suggest that its occurrence may be higher than previously reported, largely due to advances in neuroimaging techniques and increased diagnostic awareness [1,2,3,4,5,6,7,8,9,10].

One or more risk factors are detected in nearly 85% of patients with CVST (both genetic and acquired prothrombotic disorders may produce CVST). The main recognized risk factors are represented by inherited thrombophilia, young adults, women in fertile age (due to their acquired transient prothrombotic state through oral contraceptive use, pregnancy, or puerperium), infections, and malignancy [1,2,3,4,5,9,11,12]. Because CVST presents due to a multitude of predisposing factors and precipitants, it may be encountered not only by neurologists, but also by other specialists [13,14].

The pathogenesis of CVST is still incompletely known, but two main mechanisms have been proposed:

(a) Diminution of cerebrospinal fluid absorption at the level of different dural sinuses, with consecutive elevation of intracranial pressure;
(b) Increases in venular and capillary pressure (secondary to thrombosis of dural sinuses and/or cerebral veins), with subsequent brain lesions [15,16,17,18,19,20].

CVST rarely presents as a typical arterial stroke syndrome, characterized by the acute onset of focal neurological deficits associated with classical vascular risk factors. Instead, it more commonly affects younger individuals and women compared to arterial ischemic or hemorrhagic stroke [2,3,4,21].

Frequently, clinical aspects of CVST are represented by a large spectrum of non-specific neurological symptoms and signs (headaches, focal or generalized seizures, focal neurologic deficits, and/or impaired consciousness, coma included) that imitate many other disorders (e.g., arterial stroke, meningitis, encephalopathy, etc.), which leads to a management challenge [1,2,3,4,11,22,23,24,25,26]. Four main nonspecific syndromes have been noted in the literature: isolated intracranial hypertension, seizures, focal neurological abnormalities and encephalopathy. Cavernous sinus thrombosis is the only CVST example that presents a distinctive clinical aspect [1,2,3,4,13].

Different imaging methods are indispensable in diagnosing individuals with clinically suspected CVST [3,11,13]. They are represented by native and contrast-enhanced Head Computed Tomography (CT), CT Venography (CTV), Magnetic Resonance Imaging (MRI) of the Head combined with MR Venography (MRV), and, in special cases, cerebral intra-arterial angiography with venous phase imaging, direct cerebral venography, or ultrasound (US) [3,11,13,14]. Characteristics of CVST on various imaging methods can be separated into direct signs (identification of venous clot inside a cerebral vein or dural sinus), and more often indirect signs (such as cerebral edema, or cerebral venous infarct).

Acute phase therapy for CVST focuses on anticoagulation, etiological treatment for the management of risk factors, the treatment of seizures, a decrease in an elevated intracranial pressure, and the prevention of eventual cerebral herniation [3,4,22,23,24].

Unfortunately, because of its frequently misleading clinical presentation, wide spectrum of causes, neuroimaging difficulties (the overlapping signal intensities of acute thrombosis and venous flow on conventional MRI and MRV), unpredictable course, and occasional treatment problems, CVST is challenging to identify [2,3,11,17,21]. A fast diagnostic of CVST is essential, because precocious and adequate therapy plays a major role in the CVST evolution, significantly reducing the risk for acute complications and sequels [1,2,3,4]. Due to important differences in geographic, ethnic, cultural, and socio-economic features in various groups of population, there were observed significant epidemiological, etiological, and clinical variations in CVST [5,14,27].

Given the limited available data regarding CVST in Romania, further studies are needed to better characterize its demographic, etiological, clinical, and imaging profile in the local population. Uncertainties remain regarding the relative contribution of thrombophilia, infections, and female-specific risk factors in Romanian patients with CVST.

Therefore, this study aimed to explore the particularities of CVST in a Romanian monocentric cohort and to compare the observed patterns with those reported in international studies. We hypothesized that our cohort would demonstrate a distinct demographic and etiological profile compared to larger international cohorts.

Most of the information on CVST individuals was obtained from international reference cohorts such as the multicenter prospective and retrospective VENOST study (from Turkey), the International Study on Cerebral Vein and Dural Sinus Thrombosis (ISCVT) (from Europe and Latin America), data from Asia and Argentina and limited information that is available regarding CVST in Romania [5,10,14,15,27,28].

## 2. Materials and Methods

This study was designed as a retrospective observational monocentric cohort study, including all consecutive adult patients (≥18 years) diagnosed with and treated for CVST in the First Department of Neurology of the “Pius Brînzeu” Clinical Emergency County Hospital, Timisoara, Romania, over a six-year period, from 1 January 2020, to 31 December 2025. Pediatric patients were not managed within our department and were therefore excluded. In cases of repeated admissions or follow-up hospitalizations related to the same CVST episode, patients were counted only once within the cohort.

Our main objective was to evaluate, at admission in our department, their demographic, etiological, clinical and imaging data, and to assess the treatment, the length of hospital stay (LHS) and the modified Rankin Scale (mRS) and mortality at discharge and at 90 days. Our results were compared with the international literature.

All cases underwent a standardized protocol of clinical, laboratory, and neuroimaging examination. Clinical suspicion of CVST was advanced in most of our patients when they presented four major clinical syndromes in combination or isolation: intracranial hypertension syndrome (isolated headache, or associated with nausea, vomiting, papillary oedema), focal neurological deficits, seizures, and sub-acute diffuse encephalopathy (various degrees of altered consciousness). A minority of our CVST patients developed cavernous sinus thrombosis with a distinctive clinical picture: painful ophthalmoplegia.

All these clinical criteria for CVST had to be confirmed using native and contrast-enhanced Head CT, CTV, MRI of the Head combined with MRV, and, in special cases, cerebral intra-arterial angiography with venous phase imaging, in accordance with international guidelines and local availability (primarily due to the limited availability of MRV in emergency settings in Romania).

The initial CT was performed in the emergency department of our hospital, while the MRI/MRV or other imaging techniques were performed over our department in the next couple of days.

For each case, demographic data were recorded together with daily habits (smoking, alcohol intake), medical history with assessment of known risk factors for CVST: pregnancy, postpartum period, or oral contraceptive use (in women in fertile age), malignancy, infections (mainly in the facial region or ear–nose–throat infections), autoimmune diseases, brain trauma, former surgical operations.

All patients underwent a general and neurological examination, completed with ocular fundoscopy, performed either by the neurologist or by the ophthalmologist.

In addition, an extended paraclinical investigation was performed, including a complete blood count, chemistry panel, prothrombin time and activated partial thromboplastin time. These data could reveal conditions that contributed to the appearance of CVST, such as hypercoagulable state, infectious, or inflammatory diseases. Anti-platelet factor four (PF4) antibodies were searched only for suspected COVID-19 vaccination associated with CVST.

Searching for thrombophilia was performed only for patients with CVST who presented an elevated probability of severe thrombophilia (personal and/or family history of venous thrombosis, CVST in young people, or CVST in the absence of a transient or permanent risk factor). The criteria included factor V Leiden mutation, G20210A prothrombin gene mutation, MTHFR C677T and A1298C polymorphism, PAI-1 antithrombin III, serum levels of protein C, protein S, lupus anticoagulant, anticardiolipin, and anti-beta2 glycoprotein-I antibodies.

Lumbar puncture and cerebrospinal fluid (CSF) examination were performed to exclude meningitis only in peculiar cases of patients with CVST who developed isolated intracranial hypertension, patients with infection, and patients with no evident cause of infection.

Data regarding symptoms onset, type of onset, clinical spectrum, CVST localization (using neuroimaging data), and treatment were recorded and analyzed, together with clinical evolution at discharge and 90 days.

A subset of patients underwent one or more follow-up evaluations after 90 days, usually at intervals of six months, during which imaging studies and laboratory investigations were repeated and clinical status was reassessed. Consequently, the duration of observation varied between patients, depending on the number and timing of follow-up visits within the six-year study period.

Individual patient informed consent was not required due to the retrospective nature of the study and the anonymization of all data prior to analysis and publication. Approval for the study was obtained from the local hospital research ethics committee.

Descriptive statistics were applied to both categorical and numerical variables. Categorical data were summarized as frequencies and percentages, while continuous variables were described using means and standard deviations or medians, depending on data distribution. Normality of continuous variables was assessed using the Shapiro–Wilk test. Comparisons between categorical variables were performed using the Chi-square test of independence or Fisher’s exact test when expected cell counts were below 5. Differences between two groups for ordinal variables were assessed using the Mann–Whitney U test, while comparisons across more than two groups were performed using the Kruskal–Wallis test. Correlations between continuous or ordinal variables were evaluated using Spearman rank correlation. Statistical significance was set at a threshold of *p* < 0.05, corresponding to a 95% confidence level. Data analysis was conducted using MedCalc statistical software (version 23.4.5, Ostend, Belgium) licensed to our university (“Victor Babes” University of Medicine and Pharmacy, Timisoara, Romania). Graphical figures were created using Affinity V3 (version 3.2.0; Serif, West Bridgford, Nottinghamshire, UK, a subsidiary of Canva). Anatomical elements were adapted based on data from literature [7].

## 3. Results

### 3.1. Demographics

Of a total of 3819 stroke cases admitted to our department over the six-year period, 55 patients (1.2%) were diagnosed with CVST. Based on the estimated population of Timis county (~650,000 permanent residents, with an additional estimated 20,000–30,000 temporary residents, mainly students) served by our tertiary neurological center [29], the incidence of CVST was calculated at 13.75 per million per year over the observation period. Notably, the COVID-19 pandemic and the associated lockdown measures did not significantly influence the incidence. Of these, 54.5% (*n* = 30) were female, among whom 53.3% (*n* = 16) were within the reproductive age range.

The mean age at presentation for the entire cohort was 49.07 ± 18.41 years (range: 18–82). Female patients were, on average, younger than male patients, with a mean age of 46.87 ± 18.67 years compared to 51.72 ± 18.11 years in males.

Further analysis was performed by stratifying patients into two age groups: a younger group (18–55 years), comprising 63.6% of cases (*n* = 35), and an older group (>55 years), comprising the remaining patients. The younger group had a higher proportion of female patients (60%, *n* = 21), whereas in the older group, males were slightly more prevalent (55%, *n* = 11).

Within the younger group, female patients were slightly younger than male patients (36.38 ± 9.98 vs. 38.50 ± 12.11 years). In contrast, within the older group, male patients were slightly younger than female patients (68.55 ± 6.39 vs. 71.33 ± 7.23 years).

However, none of these differences reached statistical significance (independent-samples *t*-test: *p* = 0.335 for the entire cohort, *p* = 0.576 for the younger group, and *p* = 0.372 for the older group).

### 3.2. Type of Onset

Regarding clinical presentation, patients were classified according to the time from symptom onset as acute (<48 h), subacute (48 h–4 weeks), or chronic (>4 weeks). Acute cases accounted for 67.3% (*n* = 37), subacute for 20% (*n* = 11), and chronic for 12.7% (*n* = 7). Most patients (mainly those with acute onset) benefited from a short interval between symptoms onset and diagnosis, typically on the same day or within a few days. The estimated mean time to diagnosis was approximately 6 days, with a median of 2 days, reflecting a right-skewed distribution with only a few patients experiencing delayed diagnosis. No statistically significant differences in onset were observed based on gender distribution as detailed in Table 1.

In acute presentations, most patients were diagnosed on the day of admission using native cerebral CT combined with CTV, primarily due to the limited availability of MRV in emergency settings in Romania.

The data further indicates that, on average, patients with chronic CVST (52.85 ± 13.69 years) were older than those with acute or subacute presentations (50.72 ± 18.53 and 41.09 ± 19.75 years, respectively). The smaller standard deviation observed in the chronic group suggests a more homogeneous age distribution, whereas the acute and subacute groups exhibited greater variability. However, these differences did not reach statistical significance (one-way ANOVA, *p* = 0.268).

### 3.3. Neurological Syndromes

At onset, neurological syndromes encompassed several distinct clinical patterns and presented either in isolation or in combination.

Intracranial hypertension syndrome (including isolated headache) was the most frequently identified and was typically associated with a subacute or progressive onset.

Focal neurological syndromes generally presented acutely, with manifestations including motor weakness (paraparesis, hemiparesis), sensory deficits, or aphasia (especially fluent Wernicke Aphasia).

Seizures were either present at onset, representing the primary reason for emergency department presentation, or developed during hospitalization.

Encephalopathy most often had a subacute presentation, characterized by altered levels of consciousness, although a significant subset of patients presented an acute onset, usually as coma.

Cavernous sinus thrombosis typically manifested as an acute syndrome (painful ophthalmoplegia) with abrupt onset and was frequently the primary reason for presentation.

Most patients presented with isolated clinical syndromes. The most frequent was intracranial hypertension syndrome, observed in 32.7% of cases (*n* = 18), followed by focal neurological deficits in 21.8% (*n* = 12). Less common presentations included cavernous sinus thrombosis syndrome in 7.3% (*n* = 4), seizure disorder in 5.5% (*n* = 3), and encephalopathy and isolated vertigo, each in 1.8% of cases (*n* = 1).

**Figure 1:**
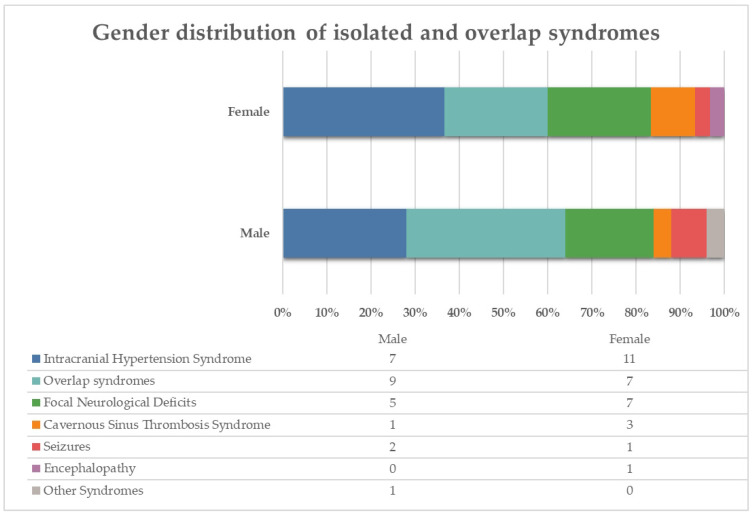
Gender distribution of different neurological syndromes at onset.

Overlap syndromes, defined as the presence of two or more concurrent clinical syndromes, were identified in 29.1% of patients (*n* = 16). The distribution of isolated and overlapping syndromes is presented in Figure 1, while the total prevalence of each syndrome is summarized in Table 1.

Focal neurological deficits, seizures and intracranial hypertension syndrome were observed with a relatively balanced distribution between isolated and overlapping presentations, while encephalopathy was associated with intracranial hypertension syndrome in the case of an acute onset, and the cavernous sinus syndrome tended to occur in isolation. An exact incidence of each syndrome is detailed in Table 1 below with no statistical significance based on gender distribution.

All patients were symptomatic, with varying degrees of severity. The mean Glasgow Coma Scale (GCS) score at admission was 13.73 ± 2.76, with approximately one-quarter of patients presenting with decreased scores (27.3%, *n* = 15), with the lowest GCS of 3 (severe coma) at presentation. The modified Rankin Scale (mRS) at admission had a mean value of 2.11 ± 1.33, with 34.6% of patients (*n* = 19) classified within grades 3–5. National Institutes of Health Stroke Scale (NIHSS) scores at admission were available for 16 patients, mainly those presenting with significant focal neurological deficits. In this subgroup, the mean NIHSS score was 8.38 ± 3.84, with a median value of 7.5, indicating moderate neurological impairment at presentation.

### 3.4. Neurological Symptoms and Signs

In terms of the clinical onset, the most common presenting complaint was headache, reported in 61.8% of cases (*n* = 34), while emesis was noted in 18.2% of patients (*n* = 10) and visual impairment (blurred or double vision) was observed in only 10.9% of cases (*n* = 6).

A considerable number of patients experienced focal and/or generalized epileptic seizures, specifically 25.5% (*n* = 14) of cases.

Comparable proportions presented with motor and sensory deficits, affecting 29.1% (*n* = 16) and 21.8% (*n* = 12) patients, respectively, while only 14.6% (*n* = 8) exhibited various forms of aphasia.

An altered mental state was observed in 27.3% of patients (*n* = 15) with varying degrees of intensity, among whom 26.7% (*n* = 4) of patients were comatose, representing 7.3% of the total lot.

Additional manifestations, including but not limited to cranial nerve palsies, painful ophthalmoplegia, cerebellar incoordination and vertigo were documented in 27.3% (*n* = 15) of the cohort.

**Figure 2:**
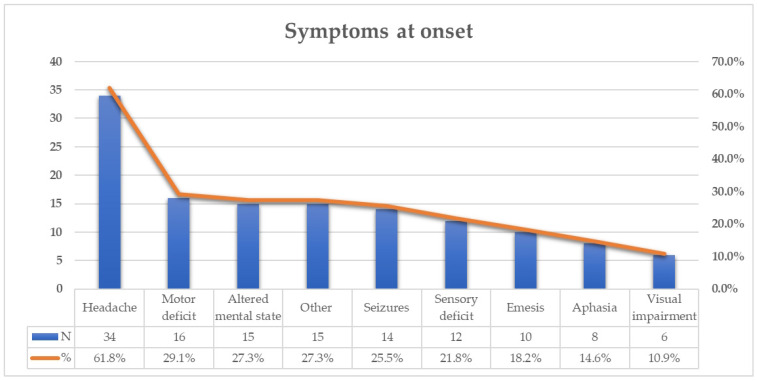
Neurological symptoms and signs at onset in CVST patients.

A graphical breakdown of neurological symptoms and signs is provided in Figure 2.

### 3.5. Neuroimaging Data

Most patients were diagnosed following presentation to the emergency department, where clinical suspicion was raised by the on-call neurologist. In contrast, incidental findings were identified in cases presenting for reevaluation in our department, in whom detailed history-taking often revealed preceding nonspecific symptoms persisting for several weeks to several months.

All patients presenting through the emergency department underwent an initial native head CT scan, followed by arterial and venous CT angiography when indicated, depending on availability and the clinical judgment of the on-call neurologist based on the initial assessment and CT findings. In many cases, the diagnosis was subsequently confirmed with MRI + MRV or established later, when initial CTV was not performed or yielded inconclusive results.

Patients that presented in the department underwent a combination of CT and CTV and/or MRI and MRV, and, in special cases, cerebral intra-arterial angiography with venous phase imaging, depending on clinical suspicion and the availability of prior imaging at the time of presentation.

All 55 patients underwent native CT, 44 cases (80%) underwent native CT and CTV, 46 patients (83.6%) underwent native CT, MRI and MRV, and 22 patients (40%) received multiple imaging modalities (CT, CTV, MRI, MRV+/-cerebral intra-arterial angiography with venous phase imaging).

The transverse sinus was the most frequently affected site, with 63.6% of patients exhibiting thrombosis, predominantly unilateral (91.4%), with bilateral involvement observed in 8.6% of cases (corresponding to 58.2% and 5.5% of the total cohort, respectively). This was followed by sigmoid sinus involvement in 54.6% of cases, occurring exclusively unilaterally. Overall, 49.1% of patients exhibited complete unilateral lateral sinus thrombosis. In a small subset of cases (5.5%), lateral sinus thrombosis was associated with contralateral transverse sinus involvement. No cases of complete bilateral lateral sinus thrombosis were identified.

The superior sagittal sinus showed a prevalence of 34.6%.

Cerebral veins thrombosis (including cortical veins, deep cerebral veins and posterior fossa veins) presented a prevalence of 34.6%. On one hand, of these, 36.8% represented isolated cerebral veins thrombosis (without associated dural sinus involvement), corresponding to 12.7% of the total cohort of 55 patients. On the other hand, cerebral veins thrombosis was associated with dural sinuses thrombosis, most commonly with lateral sinus thrombosis (57.9%) or superior sagittal sinus thrombosis (26.3%), frequently involving anastomotic veins such as the veins of Trolard and Labbé.

Cavernous sinus thrombosis was present in 12.7% of cases, occurring unilaterally in 71.4% and bilaterally in 28.6% (representing 9.1% and 3.6% of the total cohort, respectively). A statistically significant, moderate positive correlation was identified between cavernous sinus involvement and localized infections (r = 0.301, *p* = 0.025).

Straight sinus thrombosis was identified in 10.9% of patients and was consistently associated with deep cerebral veins thrombosis, and/or lateral and/or superior sagittal sinus thrombosis.

Additional sites of thrombosis were identified in 30.9% of patients, including the internal jugular veins, inferior sagittal sinus and the superior and inferior petrosal sinuses, most often in association with lateral sinus thrombosis (82.4%), as well as ophthalmic vein involvement associated with cavernous sinus thrombosis (17.7%).

**Figure 3:**
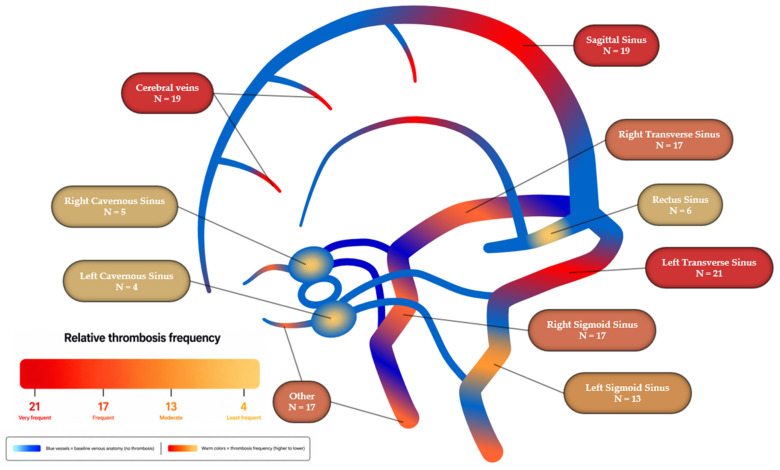
Heatmap distribution of cerebral veins and dural sinuses thrombosis in CVST patients.

Slightly more than one-quarter of patients presented with isolated sinus thrombosis, whereas the majority exhibited involvement of two or more venous territories, most commonly concurrent thrombosis of the transverse and sigmoid sinuses. A detailed breakdown of the distribution of dural sinuses and cerebral veins involvement, including laterality, is provided in Figure 3 below.

Parenchymal brain lesions were identified in 32 patients (58.2%), while 23 patients (41.8%) showed no detectable parenchymal abnormalities on neuroimaging. Among patients with parenchymal involvement, ischemic venous lesions and hemorrhagic venous lesions were equally represented, each being observed in 16 patients (29.1% of the total cohort).

### 3.6. Paraclinical Data

From a paraclinical standpoint, all patients underwent coagulation assessment, including measurements of fibrinogen, D-dimer, the international normalized ratio (INR), the prothrombin time (PT), and the activated partial thromboplastin time (aPTT).

At presentation, most coagulation parameters were elevated compared to normal reference ranges, with the exception of aPTT. Mean fibrinogen levels were 414.53 ± 169.18 mg/dL (reference range: 200–393 mg/dL), while D-dimer levels averaged 569.93 ± 995.83 ng/mL (reference range: 0–243 ng/mL). The mean INR was 1.3 ± 1.09 (reference range: 0.8–1.07), and mean PT was 14.33 ± 12.22 s (reference range: 9.4–12.5 s). In contrast, aPTT values remained within normal limits, with a mean of 29.61 ± 6.68 s (reference range: 25.1–36.5 s).

However, the elevated mean values observed for INR, PT, and aPTT are influenced by a single case of dicoumarin overdose. When considering median values, these parameters fall within normal ranges (INR: 1.09, PT: 12 s, aPTT: 28.5 s), suggesting that the overall coagulation profile was largely within normal limits, besides fibrinogen and d-dimer values.

Inflammatory syndrome was also common within the cohort, with 50.9% of patients (*n* = 28) presenting elevated CRP and ESR levels. Mean CRP values were 41.5 ± 73.34 mg/L (reference range: 0–10 mg/L), while mean ESR values were 29.65 ± 25.75 mm/h (reference range: 2–20 mm/h). However, median values were closer to the upper limit of normal (CRP: 11 mg/L; ESR: 20 mm/h), reflecting the influence of a subset of patients with markedly elevated inflammatory markers. Specifically, 14.6% of patients (*n* = 8) exhibited a pronounced inflammatory response, with CRP values > 100 mg/L and ESR values > 50 mm/h.

Spearman correlation analysis demonstrated a weak but statistically significant positive correlation between age and CRP levels (r = 0.286, *p* = 0.034), indicating that higher inflammatory responses were more frequently observed in older patients.

Genetic testing for thrombophilia was recommended for most of our patients, with almost all positive results being in the 18–55 age group. Among these, 68.6% (*n* = 24) tested positive for at least one mutation, representing 43.6% of the total cohort. Most patients with confirmed thrombophilia (83.3%, *n* = 20), representing 36.4% of the total cohort, had two or more alleles identified on genetic testing. Of the confirmed cases, 62.5% (*n* = 15) were female (27.3% of the total cohort and 50% of the female cases), while 37.5% (*n* = 9) were male (16.4% of the total cohort). However, chi-square test of independence showed no statistical significance with a *p* = 0.297.

The most frequent mutation was MTHFR C677T, identified in 70.8% of confirmed cases (*n* = 17), corresponding to 30.9% of the total cohort. Of these, 70.6% (*n* = 12; 21.8% of the cohort) were heterozygous, while 29.4% (*n* = 5; 9.1% of the cohort) were homozygous.

This was followed by PAI-1, detected in 62.5% of cases (*n* = 15), representing 27.3% of the total cohort. Among these, 25% (*n* = 6) were heterozygous, and 75% (*n* = 9; 16.4% of the cohort) were homozygous.

Factor V Leiden mutation was identified in 37.5% of cases (*n* = 9), corresponding to 16.4% of the total cohort; all cases were heterozygous.

MTHFR A1298C was present in 20.8% of cases (*n* = 5), or 9.1% of the total cohort; 80% (*n* = 4; 7.3% of the cohort) were heterozygous, while one patient presented the homozygous variant.

The least frequent mutation was prothrombin G20210A, identified in 12.5% of cases (*n* = 3), corresponding to 5.5% of the total cohort, all in heterozygous form.

Further analysis demonstrated that female patients were significantly more likely to carry the MTHFR C677T mutation in heterozygous or homozygous form compared to males. The mutation was present in all female patients tested, whereas it was identified in 55.6% of male patients (*n* = 5), yielding a statistically significant difference (χ^2^ test, *p* = 0.021). No other significant gender-based differences were observed.

### 3.7. Etiology and Risk Factors

Beyond paraclinical findings, several additional etiological risk factors were identified within the cohort. Arterial hypertension was the most frequent comorbidity, present in 54.6% of cases (*n* = 30). Metabolic risk factors were also highly prevalent, with dyslipidemia identified in 49.1% of patients (*n* = 27), followed by obesity in 32.7% (*n* = 18) and type 2 diabetes mellitus in 27.3% (*n* = 15). Smoking was reported in 30.9% of cases (*n* = 17).

Infectious conditions, considered together, were identified in 38.2% of patients (*n* = 21). Of these, paracranial localized infections were the most common in 21.8% of cases (*n* = 12), primarily represented by sinusitis of varying severity. Systemic infections were less frequent, observed in 16.4% of patients (*n* = 9), most commonly bronchopneumonia with secondary sepsis. There were also identified 4 cases which had concomitant COVID-19 viral infection.

Autoimmune disorders were present in 16.4% of cases (*n* = 9), with thyroiditis being the most frequent, accounting for 66.7% of these cases (*n* = 6; 10.9% of the total cohort), followed by systemic lupus erythematosus (*n* = 2; 3.6% of the total cohort). Hematological disorders were observed at a similar prevalence (16.4%, *n* = 9), most commonly anemia (44.4%, *n* = 4; 7.3% of the total cohort) due to iron deficiency, followed by polycythemia vera (33.3%, *n* = 3; 5.5% of the total cohort). A history of active or previous malignancy was identified in 14.6% of patients (*n* = 8).

A prior history of deep vein thrombosis and/or pulmonary thromboembolism was present in 12.7% of cases (*n* = 7). The same prevalence was observed among patients with a recent history of head trauma. Pregnancy and the puerperium accounted for 10.9% of the cohort (*n* = 6), corresponding to 20% of female patients. Oral contraceptive use was reported in 5.5% of patients (*n* = 3), representing 10% of female patients.

Risk and predisposing factors for CVST are presented in Table 2.

### 3.8. Treatment

Following admission and confirmation of CVST, all patients received anticoagulation with low-molecular-weight heparin (LMWH) throughout their hospital stay. Antiepileptic therapy was administered in 29.1% of cases (*n* = 16), including all patients with symptomatic seizures and two additional patients considered at high risk based on imaging findings.

Cerebral depletive therapy was employed in 45.5% of cases (*n* = 25), primarily using mannitol, with adjunctive use of furosemide and hypertonic saline (HTS) depending on the severity of intracranial hypertension; severe cases required combination therapy. Corticosteroids were administered in 25.5% of patients (*n* = 14), based on the clinical judgment of the treating physician.

Additional symptomatic treatments included antivertiginous agents in 9.1% of cases (*n* = 5). Supportive neurotrophic therapies, such as vitamin supplementation (B1, B6 and folic acid), were also used. Antibiotic therapy was administered in 20% of patients (*n* = 11), primarily for systemic infections, most commonly bacterial bronchopneumonia, as well as for selected cases of sinusitis.

At discharge, long-term anticoagulation, usually for 6 months, was recommended in 67.3% of patients (*n* = 37), with a preference for direct oral anticoagulants. Apixaban was the most frequently prescribed agent, used in 47.3% of patients (*n* = 26), followed by dabigatran in 14.6% (*n* = 8) and rivaroxaban in 5.5% (*n* = 3). A small proportion of patients (5.5%, *n* = 3) were advised to continue low-molecular-weight heparin (LMWH) for a short period after discharge.

Antiplatelet therapy was prescribed in selected cases with low-risk thrombophilia, being administered to 29.1% of patients (*n* = 16). Of these, two patients received dual antiplatelet therapy, while the remainder were treated with a single agent being acetylsalicylic acid in 14 cases.

### 3.9. Outcome

The mean duration of hospitalization was 12.33 ± 11.9 days (95% CI: 9.11–15.55, range: 2–78 days), the shortest stay corresponding to a chronic incidental finding.

A statistically significant difference in functional outcome (mRS) was observed across clinical syndrome groups (Kruskal–Wallis χ^2^(3) = 24.09, *p* < 0.001). Patients presenting with overlap syndromes had the highest mRS scores, indicating the worst functional outcomes. In contrast, patients with isolated intracranial hypertension syndrome demonstrated the most favorable outcomes. Cases presenting focal neurological deficits showed higher mRS scores compared to other isolated syndromes, while the remaining syndromes exhibited intermediate outcomes.

Overall therapeutic response was favorable, with reassessment at discharge showing a mean mRS score of 1.36 ± 1.78 (95% CI: 0.88–1.84). Only 16.4% (95% CI: 7.7–28.8%) of patients had moderate-to-severe disability (mRS 3–5). Three patients (5%), all male, experienced unfavorable outcomes and died during hospitalization.

**Figure 4:**
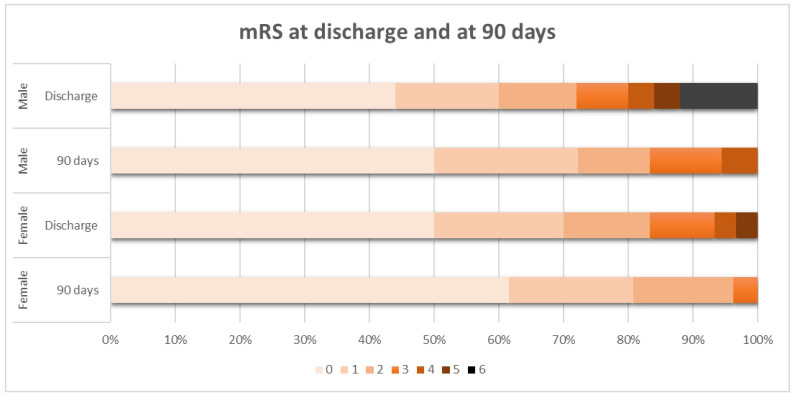
mRS data at discharge and 90-day follow-up.

Female patients showed a trend toward better functional outcomes (lower mRS scores); however, this difference did not reach statistical significance (Mann–Whitney U = 325.5, *p* = 0.374). mRS data at discharge and at 90-day follow-up are presented in Figure 4 below.

At 90 days, 44 patients (84.6% of those alive at discharge) presented for follow-up and underwent both clinical and imaging reassessment. The remaining patients were lost to follow-up or unavailable for reassessment.

Functional outcomes showed further improvement, with a mean mRS score of 0.77 ± 1.08 (95% CI: 0.48–1.06), and only 9.1% (95% CI: 3–19.9%) of patients presenting with moderate-to-severe disability (mRS 3–5). No additional deaths were recorded during the follow-up period.

Beyond global functional improvement reflected by mRS scores, most patients presenting with focal neurological deficits demonstrated partial or complete clinical recovery at discharge and follow-up, including improvement of motor deficits and aphasia. Similarly, patients presenting with seizures or altered mental status generally showed favorable neurological evolution under treatment. NIHSS was not systematically recorded in all patients and was not repeated at discharge or at 90-day follow-up, largely because many patients had non-classical CVST presentations or showed substantial clinical improvement during hospitalization.

**Figure 5:**
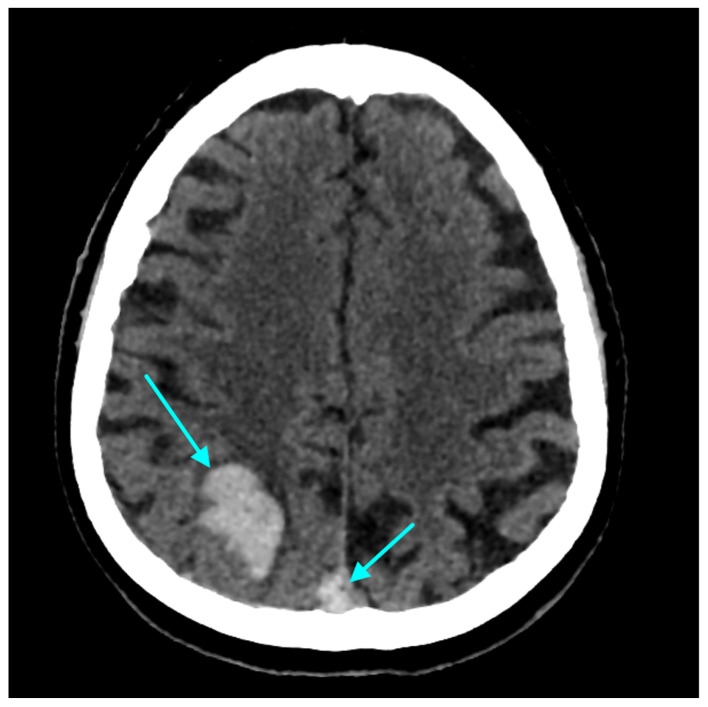
Non-contrast head CT performed in the emergency department in the acute phase demonstrated a hemorrhagic venous infarction in the right parietal lobe, with associated superior sagittal sinus thrombosis (arrows).

**Figure 6:**
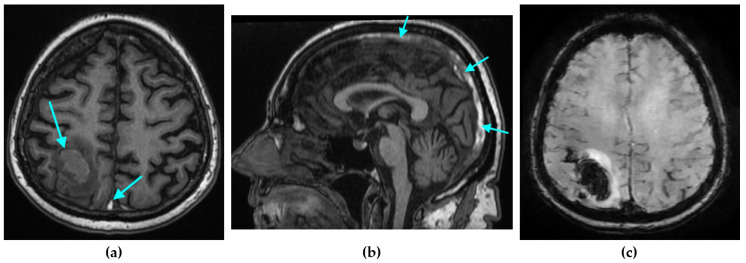
Acute-phase non-contrast brain MRI demonstrates a hemorrhagic venous infarction in the right parietal lobe and associated thrombosis of the superior sagittal sinus (arrows) with (**a**) axial T1-weighted image, (**b**) sagittal T1-weighted 3D BRAVO sequence highlighting the superior sagittal sinus and (**c**) axial 3D SWAN sequence demonstrating susceptibility changes associated with hemorrhage.

**Figure 7:**
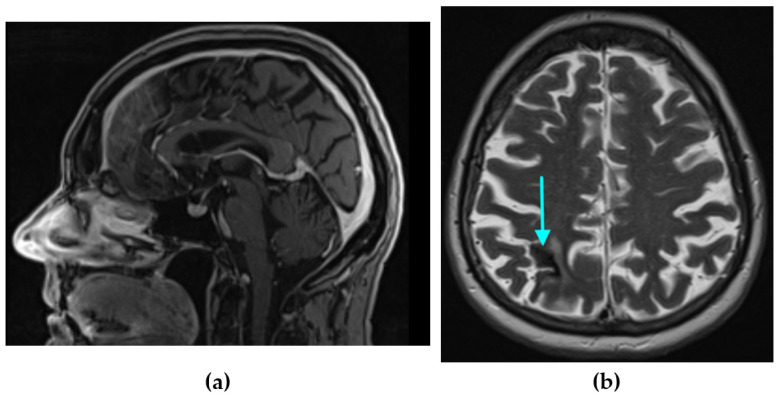
Follow-up brain MRI at 90 days demonstrates (**a**) post-contrast sagittal 3D T1-weighted MPRAGE sequence showing recanalization of the superior sagittal sinus and (**b**) axial T2-weighted TSE image showing interval reduction in the right parietal hemorrhage (arrow).

Imaging reevaluation demonstrated partial or complete recanalization of the affected venous structures and resolution of associated parenchymal lesions, particularly in a case of hemorrhagic venous infarction (Figure 5, Figure 6 and Figure 7).

At the same time, at follow-up, some patients demonstrated significant clinical improvement despite persistent chronic occlusion of the affected venous territories.

## 4. Discussion

Our study provides insight into the current state of diagnosis, management, and outcomes of CVST in Romania. Although limited to a single regional center, it benefits from an extended 6-year retrospective observation period and represents, to our knowledge, the largest reported cohort from the country.

The incidence of CVST in our cohort was slightly higher than that reported internationally, at 13.75 cases per million population per year, compared to 12.1 per million reported in a meta-analysis of over 20 studies [30]. However, our estimate remains lower than previously reported Romanian data (17.4 per million) [28]. Several limitations may affect the accuracy of our incidence calculation, including reliance on the estimated population of Timis county as the denominator and the tertiary-care referral nature of our center. Although our department represents the main neurological referral center in the region, not all CVST cases from the catchment area may have been managed exclusively at our institution, while some patients included in the cohort were referred from outside the primary service area. Therefore, the reported incidence should be interpreted as an approximate regional estimate rather than a definitive population-based incidence. The proportion of CVST among all stroke cases in our center was 1.2%, slightly above international estimates of 0.5–1% [30,31].

Gender distribution in our cohort was relatively balanced, with an approximately equal male-to-female ratio, showing only a slight predominance of female patients. This contrasts with international data, where females account for up to 75% of cases [32,33]. In our study, the younger subgroup (<55 years) showed a higher proportion of female patients (>60%), while the older group (>55 years) demonstrated a slight male predominance, in line with more recent reports [34]. However, no statistically significant differences were observed in age distribution between genders. The mean and median ages in our cohort were higher than those reported in other studies, including the BEAST study [35,36].

Regarding clinical onset, our data demonstrated a clear predominance of acute presentations, accounting for approximately two-thirds of cases. This differs from some international reports that describe a higher frequency of subacute presentations [7,37]. However, our findings are comparable to those of the VENOST study, particularly in the distribution of acute and subacute presentations, with younger patients more frequently presenting acutely compared to those with chronic onset [5].

The clinical profile of our patients was broadly consistent with previously reported data, with headache being the most commonly presented symptom. However, its prevalence in our cohort was lower (slightly over 60%) compared to rates exceeding 85% reported in the literature [38]. This lower prevalence may reflect differences in referral patterns, as patients presenting with isolated headache are less likely to be referred to or reach our neurology department, rather opting for a simple consultation outside the hospital.

Focal neurological deficits were comparable to those described in prior studies, with motor deficits observed in 29.1% of our patients, within the reported range of 19.1–39% [39]. The prevalence of aphasia was slightly lower in our cohort (approximately 14%) compared to reported values of 19–24% [39,40,41]. One limitation of our data is that NIHSS scores were available only for a subset of patients with more prominent focal neurological deficits and were recorded only at admission. Therefore, NIHSS could not be used to assess longitudinal neurological evolution across the cohort.

Seizures were somewhat less frequent in our study, occurring in approximately 25% of cases compared to 30–40% reported in the literature, including ISCVT [14]. In contrast, sensory deficits were more common in our cohort (>20%) compared to approximately 5% reported in the same study [14].

The frequency of altered mental status was comparable to that reported in recent studies, including those published in the *European Stroke Journal* [42]. In contrast, we observed a lower prevalence of visual impairment, papilledema, and cranial nerve palsies compared to previously discussed reports [14,39,40,42].

All patients underwent initial evaluation with non-contrast head CT, in accordance with international recommendations [43,44]. Diagnostic confirmation was subsequently achieved using CTV or MRI combined with MRV, modalities shown to provide the highest diagnostic accuracy for CVST [45]. In our cohort, the majority of patients (>80%) underwent MRI with MRV for confirmation, a rate comparable to that reported in other larger multicenter studies [5,7].

We performed a comparative analysis of sinus localization and etiological factors against both international and regional cohorts (Table 3). 

In our cohort, the transverse and sigmoid sinuses were the most frequently affected structures (63.6%), a finding consistent with several published series. This prevalence is comparable to that reported in studies such as VENOST (73.4%), as well as in the Argentinian cohort and the FPCCVT registry (50–80%) [5,10,46]. Sigmoid sinus involvement (54.6%) in our cohort also falls within the range described in the literature [47].

In contrast, the involvement of the superior sagittal sinus ranked second in our cohort (34.6%) and was lower than that reported in several international studies, including ISCVT (62%), FPCCVT (48.3%), and the Argentinian cohort (46%) [7,10,14,46]. However, similar or even lower prevalences have been reported in VENOST (39.8%) and other regional studies, including Romanian data (~20%) [5,15].

These differences reflect a broader variability in the literature, with some studies identifying lateral (transverse and/or sigmoid) sinus involvement as the most frequent [48,49,50], while others report the superior sagittal sinus as the predominant site [51,52].

Straight (rectus) sinus thrombosis was observed in 10.9% of our patients, aligning with the literature-reported values ranging from approximately 10% to 18% [5,14,27,53].

Cavernous sinus thrombosis was more frequently identified in our cohort (12.73%) compared to most published data, where it remains a relatively rare presentation, typically reported in 1–3% of cases [14,46]. This discrepancy may reflect regional differences in etiology, particularly the higher prevalence of infection-related cases. In support of this, we identified a statistically significant positive correlation between cavernous sinus involvement and infection, reinforcing the known association between infectious processes and this localization. Similar or even higher rates have been reported in Romania and in developing countries [54,55,56].

Cerebral vein involvement, including cortical, deep, and posterior fossa veins, was observed in 34.6% of our cases. Although direct comparison is limited due to differences in reporting, this prevalence appears somewhat higher than the combined rates typically reported for cortical and deep venous thrombosis, which generally range between 20% and 30% [57,58,59,60].

The distribution of parenchymal lesions in our cohort was consistent with the previously published literature, with approximately 42% of patients showing no detectable parenchymal lesions, while the remaining patients demonstrated either ischemic or hemorrhagic venous lesions. Hemorrhagic venous infarction represented a substantial proportion of parenchymal involvement, reflecting the characteristic venous congestion and blood–brain barrier disruption associated with CVST [5,46].

From an etiological perspective, thrombophilia was markedly more prevalent in our cohort (68.6%) compared to other studies, including ISCVT (34.1%) and VENOST (26.4%), although similar values have been reported in other regional registries [5,14,15,61,62,63]. This finding may suggest a higher prevalence of thrombophilia among CVST patients in our population.

However, this difference may also be explained by the extensive use of genetic testing in our cohort, particularly among younger patients, who were significantly more affected than older individuals in our analysis, leading to increased detection rates [64,65].

Additionally, a statistically significant sex-related difference was observed, with the MTHFR C677T mutation being more prevalent in female patients compared to males in our cohort. This finding contrasts with most published data reporting no significant sex-based differences and may be influenced by the relatively limited cohort size. Furthermore, recent evidence suggests that MTHFR polymorphisms are no longer considered independent direct causes of CVST, but rather secondary contributing factors, mainly through their association with hyperhomocysteinemia [66].

Infections were more commonly identified in our cohort (38.2%) compared to international data [5,14,46]. However, direct comparison is limited by differences in classification, particularly regarding the grouping of local and systemic infections. Notably, our findings are more comparable to those reported in regional or single-center studies [15,67]. An emerging area of interest is the potential association between recent COVID-19 infection and CVST, as well as other thromboembolic events. In our cohort, a small number of cases were observed in this context, consistent with findings reported in the literature [68,69].

Similarly, malignancy and hematological disorders were more frequently observed in our cohort (14.6% and 16.4%, respectively) compared to most published series [70,71,72,73,74]. However, these comparisons should be interpreted with caution, as some literature values are estimated and categories are not uniformly defined across studies. In addition, the relatively small sample size of our cohort compared to large registries such as ISCVT and FPCCVT may further limit direct comparability.

Autoimmune conditions were also more prevalent in our population (16.4%) compared to ISCVT but were closer to values reported in FPCCVT [14,46]. This difference may partly reflect variations in classification, particularly regarding the inclusion of antibody-mediated conditions and the use of estimated values in comparative data [75,76,77].

Notably, the spectrum of autoimmune diseases differed between cohorts. While Behçet’s disease has been reported with a relatively high prevalence in studies such as VENOST [5] and other regional cohorts [78,79], no such cases were identified in our population. In contrast, thyroid-related autoimmune conditions were more frequently observed in our cohort, in line with findings from other reports highlighting their association with CVST [80,81].

Female-specific risk factors showed a heterogeneous distribution. While pregnancy and puerperium rates in our cohort (20%) were comparable to those reported in ISCVT and other studies [5,14,82,83,84], the prevalence of oral contraceptive use was notably lower than that observed in international cohorts [85,86,87]. This difference likely reflects regional variations in contraceptive practices and population characteristics, as Romania has one of the lowest rates of contraceptive use in the European Union [88,89].

An important observation is that, despite the higher prevalence of malignancy, infection, and autoimmune conditions in our cohort, no statistically significant differences were identified in their distribution across sex or predefined age groups.

However, from a paraclinical perspective, a statistically significant correlation was observed between inflammatory markers and patient age, indicating a more pronounced inflammatory response in older patients with CVST. This finding is supported by previous reports demonstrating a two- to four-fold increase in inflammatory response in older individuals compared to younger patients, which may contribute to vascular pathology [90,91,92,93].

All patients in our cohort received anticoagulation in accordance with the current literature recommendations [94,95], with-low molecular-weight heparin administered throughout the hospitalization period.

Antiepileptic medication was used in 29.1% of patients, primarily in those presenting with seizures, as well as in selected individuals considered at high risk. This is consistent with the literature data reporting usage rates ranging from 20% to 40% [96,97,98,99].

Cerebral depletive therapy was administered in 45.5% of cases, most commonly using mannitol. This finding is in line with the available literature, where the use of osmotherapy is estimated to range from approximately 20% to as high as 60%, depending on the severity of intracranial hypertension [100,101,102,103].

At discharge, long-term anticoagulation was recommended in 67.3% of patients, typically for a duration of approximately 3 to 6 months in most cases, depending on the underlying etiology (permanent or transitory risk factors). Direct oral anticoagulants (DOACs) were preferred, with apixaban being the most frequently prescribed agent, followed by dabigatran and rivaroxaban. This pattern is consistent with recent literature demonstrating increasing use of DOACs in CVST, with reported usage rates ranging from 40% to 70% [7,104,105,106]. A small proportion of patients (5.5%) continued low-molecular-weight heparin, in line with reported practice in selected cases [107,108].

The mean duration of hospitalization in our cohort (12.33 ± 11.9 days) is in line with previously reported data, with most studies describing average lengths of stay between approximately 10 and 16 days, although shorter durations have been reported in large administrative datasets [46,109,110,111,112].

Patient outcomes were assessed using the modified Rankin Scale (mRS), in accordance with international practice [113]. Our results were comparable to those reported in other registries, with 70–80% of patients achieving favorable outcomes (mRS ≤ 2) at discharge and over 90% at 90-day follow-up. These findings are consistent with international data demonstrating similar recovery rates in CVST patients [14,114,115,116]. These findings suggest that, despite the somewhat distinct etiological profile observed in our local population, the case management and treatment approaches applied in our center proved effective, resulting in functional outcomes comparable to those reported by larger international cohorts and tertiary-care institutions.

As expected, statistical analysis showed worse outcomes in patients with the involvement of multiple sinuses and cerebral veins, as well as in those presenting with overlapping clinical syndromes compared to isolated presentations. These observations are in line with findings reported in the literature [40,117,118,119].

A sex-related difference was also observed, with female patients demonstrating a trend toward better functional outcomes compared to males. Although this difference did not reach statistical significance in our cohort, comparable trends have been described in the literature, suggesting a potential influence of sex on CVST prognosis [14,48,120].

Furthermore, poorer functional outcomes were more frequently observed in patients presenting significant focal neurological deficits associated with higher NIHSS scores and lower GCS values at admission, as well as in those with more extensive cerebral parenchymal lesions, particularly hemorrhagic venous infarctions. Although these observations were primarily descriptive and no dedicated multivariate prognostic statistical analysis was performed due to the relatively limited cohort size, the observed trends are consistent with previously reported predictors of unfavorable outcome in CVST.

Interestingly, follow-up imaging findings at 3 months did not consistently correlate with clinical presentation or functional outcomes. Although most patients demonstrated partial or complete recanalization of the affected venous structures, some exhibited persistent or chronic occlusion despite marked clinical improvement and favorable mRS scores. This highlights the known dissociation between radiological and clinical recovery in CVST [14,59,121].

This study has several limitations, including its retrospective design, single-center setting, and relatively small sample size, which may limit the generalizability of the findings. As a tertiary neurological referral center, our cohort may also be affected by referral and selection bias, with a potential overrepresentation of more severe or complex CVST cases. Additionally, some clinical and follow-up data were incomplete due to loss to follow-up and the non-uniform retrospective documentation of neurological scales. Variability in diagnostic imaging availability, follow-up imaging protocols, and therapeutic approaches between patients may have further influenced outcome assessment. Finally, the relatively limited cohort size precluded robust multivariate prognostic statistical analysis.

## 5. Conclusions

Cerebral venous thrombosis remains a highly heterogeneous condition, with considerable variability in clinical presentation, localization, and underlying etiologies. In our Romanian cohort, thrombophilia and infections represented particularly important contributing factors, while traditional female-specific risk factors appeared less prominent compared to larger international studies. We also observed a relatively increased prevalence of cavernous sinus thrombosis, frequently associated with localized infections.

These findings highlight the importance of performing comprehensive etiological evaluation, including genetic, immunological, and infectious assessment, within a multidisciplinary framework. Despite the distinct etiological profile observed in our cohort, functional outcomes remained favorable and comparable to those reported in major international registries, emphasizing the importance of early diagnosis and individualized therapeutic management. The observed dissociation between radiological and clinical recovery further supports a patient-centered approach to follow-up and outcome assessment.

## Figures and Tables

**Table 1:** Gender-based comparison of clinical syndrome and onset prevalence of CVST patients.

		Female		Male		Total		*p*
		*n*	%	*n*	%	*n*	%	
Age group	All	30	100	25	100	55	100	0.340
	18–55	21	70	14	56	35	63.6	0.283
	>55	9	30	11	44	20	36.4	0.283
Type of onset	Acute	20	66.7	17	68	37	67.3	0.916
	Subacute	7	23.3	4	16	11	20	0.498
	Chronic	3	10	4	16	7	12.7	0.689
Syndrome prevalence	ICH	21	70	13	52	34	61.8	0.171
	Focal deficits	11	36.7	13	52	24	43.6	0.254
	Encephalopathy	6	20	9	36	15	27.3	0.185
	Seizures	6	20	8	32	14	25.5	0.309
	Painful ophthalmoplegia	3	10	4	16	7	12.7	0.689
	Overlap	7	23.3	9	36	16	29.1	0.303

**Table 2:** Risk and Predisposing factors for CVST.

Factor		Age Group				*p*
		18–55		>55		
		*n*	%	*n*	%	
	Obesity	11	31.4	7	35	0.786
	Smoking	10	28.6	7	35	0.620
	Malignancy	4	11.4	4	20	0.443
	Trauma	4	11.4	3	15	0.696
	Venous thrombosis	3	8.6	4	20	0.242
	Inflammatory syndrome	15	42.9	13	65	0.114
Infection	All	12	34.3	9	45	0.431
	Paracranial	8	22.9	4	20	1
	Systemic	4	11.4	5	25	0.261
	Autoimmune	7	20	2	10	0.462
	Hematological	5	14.3	4	20	0.709
	Pregnancy and puerperium	6	17.1	0	0	0.076
	Oral contraceptives	3	8.6	0	0	0.293
	Thrombophilia	23	65.7	1	5	<0.001

**Table 3:** Comparative data of localization and etiological factors from international registries.

	Local	ISCVT	VENOST	FPCCVT	M. Alet et al.	R. Balasa et al.
Location	Romania	International	Turkey	France	Argentine	Romania
Transverse	63.6	40–80 *	73.4	50–80 *	70	44.4
Sigmoid	54.6	40–80 *	39.8	50–80 *	46	11.1
Superior Sagittal	34.6	62	38.9	48.3	44	20
Straight	10.9	18	n/a	13.4	18	2.2
Cavernous	12.7	1.3	1.7	0.4	n/a	15.6
Thrombophilia	68.6	34.1	26.4	22.1	28	59.5
Infection	38.2	12.3	8.1	4	4	32.4
Malignancy	14.6	7.4	5.2	4–5 *	15	n/a
Hematological	16.4	12	1–2 *	10–12 *	n/a	n/a
Autoimmune	16.4	5–7 *	10–15 *	8–10 *	n/a	n/a
Pregnancy & Puerperium	20	20.1	27.8	3.2	12	27.3
Contraceptives	10	54.3	13.9	71	44	13.6

Values marked with an asterisk (*) represent estimates derived from published data and should be interpreted with caution, as categories are not uniformly defined across studies and may not be directly comparable.

## Data Availability

The datasets are not publicly available. Anonymized data may be provided upon request from Razvan Bertici.

## Abbreviations

The following abbreviations are used in this manuscript:

CVST	Cerebral venous sinus thrombosis
DOAC	Direct oral anticoagulant
mRS	Modified Rankin Scale
US	Ultrasonography
LHS	Length of hospital stay
ICH	Intracranial Hypertension
CT	Computed Tomography
CTV	Computed Tomography Venography
MRI	Magnetic resonance imaging
MRV	Magnetic Resonance Venography
NIHSS	National Institute Stroke Scale
